# Correlation analysis of metabolic parameters influencing postoperative tendon healing after Achilles tendon rupture repair

**DOI:** 10.3389/fmed.2026.1829337

**Published:** 2026-06-30

**Authors:** Weixin Ye, Zhenchen Hou, Chang Liu, Zhenlong Liu

**Affiliations:** 1 Department of Sports Medicine, Peking University Third Hospital, Institute of Sports Medicine of Peking University, Beijing, China; 2 Peking University Third Hospital Chongli, Beijing, China; 3 Beijing Key Laboratory of Research and Translation for Drugs and Medical Devices in Precision Diagnosis and Treatment of Sports Injuries, Beijing, China; 4 Engineering Research Center of Sports Trauma Treatment Technology and Devices, Ministry of Education, Beijing, China

**Keywords:** Achilles tendon rupture, hyperuricemia, metabolic dysregulation, postoperative healing, signal-to-noise quotient (SNQ)

## Abstract

**Background:**

Postoperative healing quality critically determines functional recovery following Achilles tendon rupture (ATR) repair. Poor healing often leads to postoperative re-rupture. While mechanical factors influencing healing are well-studied, the role of metabolic disease remains unclear. Emerging evidence suggests metabolic disorders may impair tendon repair, yet their impact on early postoperative outcomes is underexplored.

**Purpose:**

This study hypothesized that metabolic disease (such as hyperuricemia and dyslipidemia) negatively affect early tendon healing after ATR repair. We aimed to identify key metabolic predictors of delayed healing to guide optimal management of perioperative metabolic parameters.

**Methods:**

Sixty patients with acute ATR undergoing surgical repair were included. Preoperative serum levels of uric acid (UA), triglycerides (TG), total cholesterol (TC), high−/low-density lipoprotein (HDL-C, LDL-C), and total bilirubin (TBIL) were measured. Postoperative signal-to-noise quotient (SNQ) values were calculated from T2-weighted MRI at 3 months. In preliminary analyses, Spearman’s rank correlation and multivariate linear regression were conducted to assess associations between six metabolic biomarkers and SNQ, identifying significant metabolic markers. Subgroup analyses subsequently stratified patients into cohorts based on these determinants, with Mann–Whitney U test comparing intergroup SNQ differences.

**Results:**

Serum UA showed a significant positive correlation with SNQ (*ρ* = 0.400, *p* = 0.002). Multivariate linear regression confirmed UA as an independent predictor of elevated SNQ (*β* = 0.394, *p* = 0.006). Lipid markers (TC, TG, HDL-C, LDL-C) and TBIL demonstrated no significant associations. Subgroup analyses compared SNQ levels between hyperuricemic patients and normouricemic controls. Hyperuricemia patients exhibited markedly higher median SNQ values than controls (14.57 vs. 7.27, *p* < 0.001), indicating delayed healing.

**Conclusion:**

Hyperuricemia is a significant metabolic factor impairing early ATR healing, highlighting the clinical utility of preoperative UA screening for personalized rehabilitation. Perioperative uric acid control is recommended for hyperuricemic patients to optimize Achilles tendon healing outcomes.

## 1 Introduction

Achilles tendon rupture (ATR) is a common sports injury in the field of sports medicine, with a steadily increasing incidence over recent decades (1). Surgical repair has been the primary treatment for acute ATR. Despite advancements in surgical techniques, approximately 20% of patients still experience adverse outcomes, including delayed union, nonunion, and re-rupture (2). Subsequent surgical interventions following re-rupture are associated with significantly compromised healing efficacy. Consequently, postoperative healing quality critically determines functional recovery and the capacity for return to sports (3). While traditional research has focused on mechanical factors (e.g., suture techniques, early mobilization) influencing healing, emerging evidence suggests that systemic metabolic dysregulation may profoundly impair tendon repair processes and functional restoration.

Previous studies have identified metabolic disorders—including obesity, hyperuricemia, hypercholesterolemia, and diabetes—as risk factors for ATR (4). Clinical investigations have demonstrated that hyperuricemia serves as an independent risk factor for ATR, with hyperuricemia patients exhibiting significantly higher rupture susceptibility compared to healthy individuals (5, 6). Dyslipidemia, diabetes, and other metabolic disturbances further exacerbate tendon degeneration by disrupting cellular metabolism, inflammatory responses, and collagen synthesis (4). Notably, Liang et al. demonstrated that uric acid dose-dependently suppresses the activity of tendon stem/progenitor cells (TSPCs), thereby compromising structural integrity and negatively affect tendon vulnerability (7). Such metabolic factors may also impair tendon replenishment and reconstruction, ultimately undermining postoperative healing outcomes.

Existing literature predominantly focuses on risk factors for ATR, with limited exploration of the relationship between systemic metabolic function and postoperative tendon healing. To date, no consensus exists regarding the determinants of post-ATR healing efficacy. This study pioneers the integration of metabolic profiling into ATR recovery analysis, utilizing the signal-to-noise quotient (SNQ) derived from MRI T2-weighted imaging as an early-stage healing biomarker. We hypothesize that Metabolic disease (such as hyperuricemia and dyslipidemia) exert detrimental effects on early postoperative healing in ATR patients. This study aims to provide novel insights for clinical research on rehabilitation strategies and therapeutic interventions following ATR repair.

## 2 Materials and methods

### 2.1 Study design and participants

This single-center retrospective study included 60 patients diagnosed with Achilles tendon rupture (ATR) who underwent surgical repair at our hospital between April 2015 and May 2024. The inclusion criteria were as follows: (i) Closed acute Achilles tendon rupture in patients aged 20–50 years with high athletic demands, treated with open repair within 2 weeks of injury; (ii) Preoperative blood biochemical testing; (iii) Preoperative MRI confirmation and postoperative MRI re-examination at 3 months. Exclusion criteria comprised: (i) Patients with direct sharp trauma (open Achilles tendon rupture) (ii) Patients receiving systemic or local corticosteroid/quinolone therapy. This retrospective study was approved by the Institutional Review Board of Peking University Third hospital (IRB M20250265).

### 2.2 Surgical procedure

Open repair was performed for acute Achilles tendon rupture. The procedure was conducted under epidural anesthesia with the patient in the prone position. The affected limb was routinely disinfected and draped. A 5–8 cm longitudinal incision was made along the medial or lateral border of the Achilles tendon. The skin, subcutaneous tissue, and paratenon fascia were sequentially incised to fully expose the ruptured tendon. Hematoma and necrotic tissue at the rupture site were evacuated, followed by trimming of tendon ends to fresh surfaces while preserving healthy tendinous tissue to enhance suture strength. Tension adjustment was achieved by positioning the knee in 30° flexion and the ankle in 20°–30° plantarflexion to minimize post-repair tension and prevent re-rupture. High-strength sutures (Ethibond (Ethicon, Johnson & Johnson, Somerville, NJ, USA)) were applied using overlapping mattress sutures to ensure precise approximation of tendon ends. The incision was closed in layers: absorbable interrupted sutures were used for the paratenon and subcutaneous layers to reduce adhesion risk, while subcuticular or interrupted sutures were applied for skin closure.

### 2.3 Rehabilitation protocol

Postoperative immobilization involved a long-leg cast maintaining the ankle in 20°–30° plantarflexion for 4–6 weeks with strict non-weight bearing. Isometric quadriceps contractions and toe mobilization were performed during cast immobilization to prevent muscle atrophy. At 6–12 weeks postoperatively, the cast was replaced with an adjustable brace to gradually restore ankle range of motion and partial weight-bearing. Resistance training and balance exercises were initiated after 12 weeks to promote complete functional recovery.

### 2.4 Detection methods

Fasting venous blood samples were collected from patients one day preoperatively. Serum levels of six metabolic markers - uric acid (UA), triglycerides (TG), total cholesterol (TC), high-density lipoprotein (HDL-C), low-density lipoprotein (LDL-C), and total bilirubin - were measured using a fully automated biochemical analyzer (Modular 7,600, Hitachi, Tokyo, Japan).

### 2.5 Magnetic resonance imaging assessments

**Figure 1:**
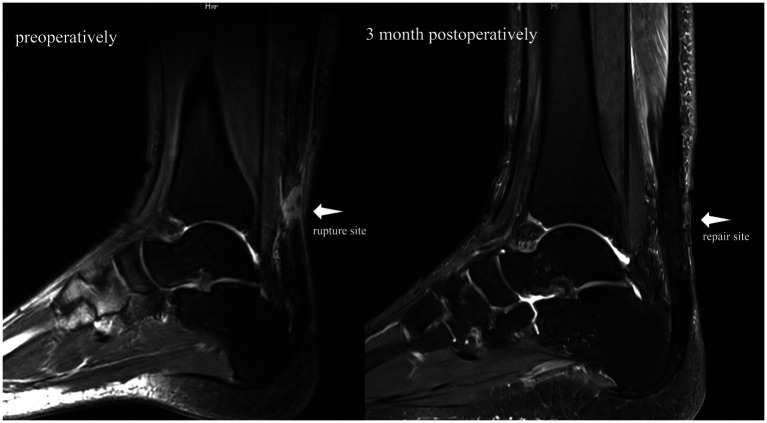
Comparison of ankle MRI images preoperatively and at 3 months postoperatively. Arrows indicate the rupture site (left) and the repair site (right), respectively.

**Figure 2:**
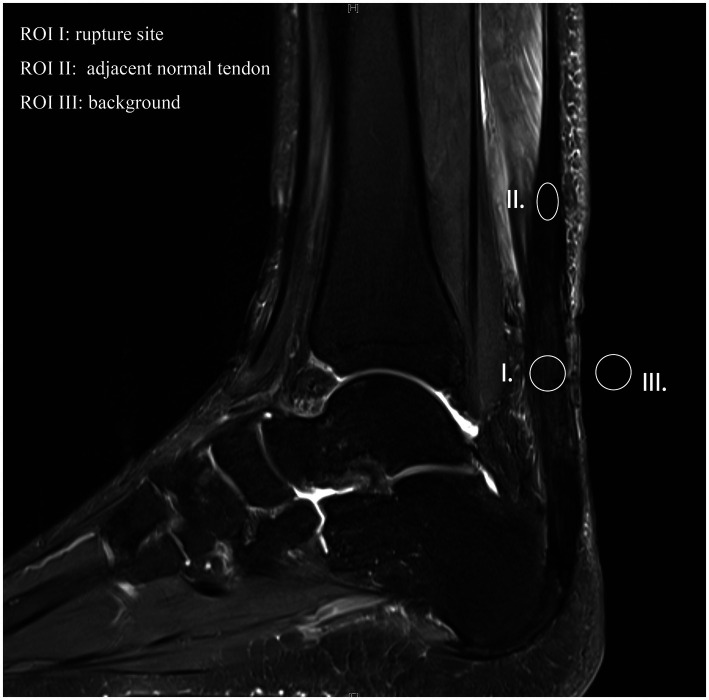
Schematic diagram of three regions of interest (ROIs) on sagittal ankle MRI. ROI I: rupture site; ROI II: adjacent normal tendon; ROI III: background.

Patients underwent MRI examinations preoperatively and 3 months postoperatively. The patients’ ankle was immobilized in neutral position and scanned with a 3.0-T MRI system (Discovery MR 750 W; GE Medical Systems, USA). Axial and sagittal T2-weighted fast spin-echo sequences were acquired with the following parameters: repetition time (TR) = 2,200 ms, echo time (TE) = 30 ms, slice thickness = 3 mm. Fat suppression was applied to enhance tissue contrast. Figure 1 shows a comparison of the patient’s preoperative and 3-month postoperative status. On postoperative 3-month sagittal T2WI images, regions of interest (ROIs) were selected for signal intensity measurement to calculate signal-to-noise quotient (SNQ). Three elliptical ROIs were defined: the rupture site, adjacent normal tendon, and background region (Figure 2). The lengths from the insertional site to the rupture site were measured on the preoperative MRI images. The repaired sites were confirmed on postoperative MRI images according to the previous measured data. The ROI at the rupture site encompassed the hyperintense region on sagittal images (ROI I). The adjacent normal tendon ROI was placed 3 cm proximal to the rupture site within intact tendon fibers (ROI II). The background ROI was positioned at the same level as the rupture site, approximately 1 cm away (ROI III). Measurements were performed twice by two blinded radiologists with an interval of 1-week independently, with the average value used as the final result. The signal-to-noise quotient (SNQ) was calculated using the following formula:

$$\begin{array}{l} \mathrm{SNQ}=(\text{SIGNA}L_{\text{Rupture Site}}-\text{SIGNA}L_{\text{Normal Tendon}})\\ /\text{SIGNA}L_{\text{Background}} \end{array}$$

Higher SNQ values indicate increased water content, suggesting poorer healing status of the Achilles tendon.

ROI I: rupture site, ROI II: adjacent normal tendon, ROI III: background.

### 2.6 Statistical analysis

Data preprocessing included normality testing and SNQ logarithmic transformation. The original SNQ values demonstrated non-normal distribution as confirmed by Shapiro–Wilk test (W = 0.89, *p* < 0.001), thus natural logarithmic transformation was applied to improve distribution morphology: log-transformed SNQ = ln (SNQ). The transformed data passed normality verification (W = 0.979, *p* = 0.404). Spearman’s rank correlation was used to assess associations between original SNQ values and six metabolic markers. Multiple linear regression analyzed relationships between metabolic indices and log-transformed SNQ, with variance inflation factors (VIF < 5) excluding multicollinearity interference. Significant biochemical markers were determined based on the results of correlation analysis and multiple linear regression analysis. Further subgroup analysis categorized patients into disease-positive and disease-negative cohorts according to established diagnostic criteria for metabolic disorders, with Mann–Whitney U test comparing intergroup SNQ differences. The ICC for the measurements of the two radiologists was calculated using a two-way random effects model, absolute agreement, for single measurement. All analyses were performed with R 4.4.3 with significance level set at *p* < 0.05.

Among 60 patients, 15 underwent emergency biochemical testing and consequently lacked four metabolic markers data: total cholesterol, triglycerides, HDL, and LDL. Comparison of baseline characteristics between missing and complete groups (Supplementary Table S1) showed a significant difference only in age (*p* = 0.016), but not in uric acid or SNQ. As missingness depended on an observed variable (age), the data were considered missing at random (MAR) assumptions. We used the MICE (multivariate imputation by chained equations) method of multiple multivariate imputation in R. The missing variables were imputed via Predictive Mean Matching (PMM), generating 20 imputed datasets (m = 20) with a maximum of 10 iterations (maxit = 10). We averaged estimates of the variables to give a single mean estimate and pooled the result according to Rubin’s rules in the multivariate linear regression analyses.

## 3 Result

The analysis results consist of two parts: preliminary analysis and subgroup analysis.

### 3.1 SNQ preliminary analysis

**Figure 3:**
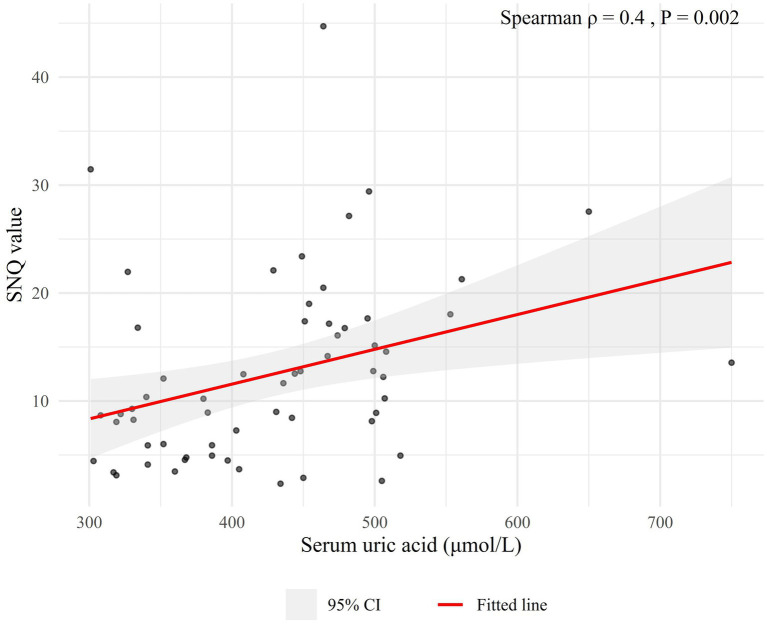
Scatter plot of the correlation between serum uric acid and SNQ values. The red line represents the fitted line, and the shaded area indicates the 95% confidence interval.

**Figure 4:**
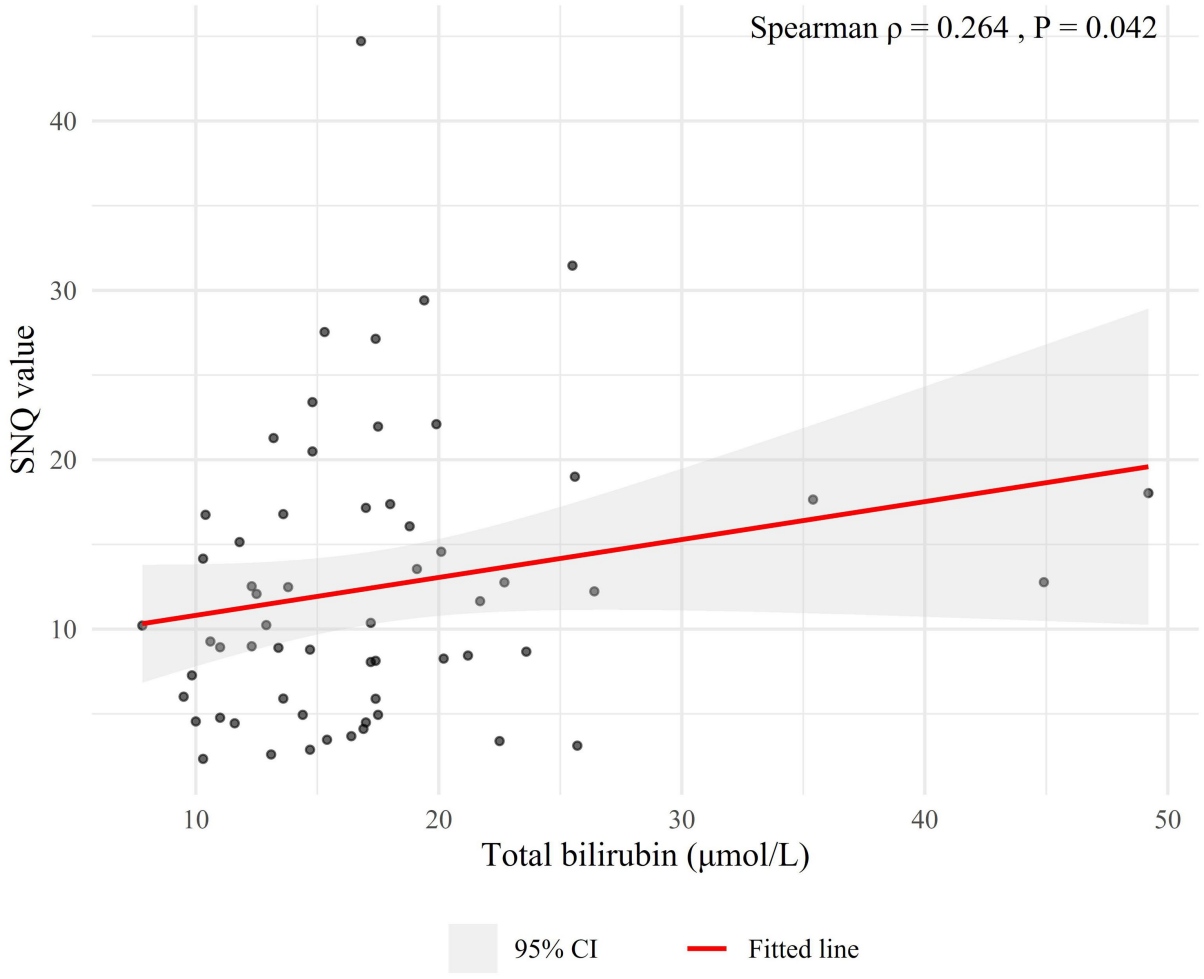
Scatter plot of the correlation between total bilirubin and SNQ values. The red line represents the fitted line, and the shaded area indicates the 95% confidence interval.

The ICC index for the interobserver reliability of the SNQ between the two surgeons was 0.95(0.922, 0.971) respectively, indicating good inter- and intraobserver reliability. We first conducted the analysis of SNQ and six metabolic markers. This part included Spearman’s rank correlation analysis and multiple linear regression analysis, with the purpose of identifying significant factors among the six metabolic markers. Spearman’s rank correlation analyses were performed in patients with complete datasets to evaluate relationships between postoperative SNQ and six metabolic biomarkers (Table 1). Spearman’s rank correlation analysis revealed significant positive associations between SNQ values and serum uric acid (*ρ* = 0.400, *p* = 0.002), suggesting that elevated UA levels may associate with poor tendon healing outcomes. Similarly, total bilirubin (TBIL) showed a positive correlation with SNQ measurements (ρ = 0.264, *p* = 0.042). Scatter plots showing the relationships of uric acid and total bilirubin with SNQ values are presented in Figures 3, 4, respectively. However, with the numbers available, no significant difference could be detected between SNQ values and the following markers, including Total cholesterol, Triglycerides, High-density lipoprotein, Low-density lipoprotein.

**Table 1:** Spearman’s correlation analysis of SNQ with metabolic markers.

Metabolic marker	Correlation coefficient (ρ)	*p*-value	Sample size (*N*)
Uric acid	0.400*	0.002	60
Total bilirubin	0.264*	0.042	60
Total cholesterol	0.044	0.774	45
Triglycerides	0.119	0.435	45
HDL	0.019	0.900	45
LDL	0.021	0.894	45

Significant at α = 0.05 (two-tailed).

To evaluate the independent effects of metabolic markers on SNQ values while controlling for confounding factors such as sex, age and BMI, we constructed a multivariate linear regression model with Post-imputation data. Total cholesterol (TC) was excluded due to severe multicollinearity (VIF = 11), while other variables exhibited acceptable collinearity thresholds (VIF < 5). The final model is presented in Table 2 (R^2^ = 0.234, adjusted R^2^ = 0.114).

**Table 2:** Multivariable linear regression results for log-transformed SNQ.

Predictor	Unstandardized β (95% Cl)	S. E	Standardized β	t-value	*p*-value	VIF
Constant	2.135(−1.113, 5.384)	1.605	–	1.331	0.191	–
Uric acid	0.003(0.001, 0.005)	0.001	0.394	2.891	0.006	1.17
Triglycerides	0.064(−0.156, 0.284)	0.109	0.106	0.586	0.561	1.78
HDL	−0.851(−2.099, 0.399)	0.608	−0.266	−1.398	0.173	1.54
LDL	0.184(−0.184, 0.554)	0.182	0.175	1.015	0.317	1.50
Total bilirubin	0.016(−0.010, 0.043)	0.013	0.179	1.253	0.217	1.26
Age	−0.001(−0.026, 0.025)	0.013	−0.007	−0.051	0.959	1.30
BMI	−0.032(−0.125, 0.061)	0.046	−0.132	−0.692	0.493	2.02
Sex	−0.363(−1.563, 0.838)	0.596	−0.094	−0.608	0.546	1.50

Among the six metabolic markers analyzed, only serum uric acid (UA) was identified as an independent positive predictor of log-transformed SNQ values (*β* = 0.394, *p* = 0.006), indicating a significant association between elevated UA levels and poor postoperative healing outcomes. With the numbers available, no significant difference could be detected between log-transformed SNQ and the following markers: Triglycerides, High-density lipoprotein, Low-density lipoprotein, Total bilirubin, Age, BMI, sex. This result matched the multiple linear regression result using unimputed complete-case data (Uric Acid: *β* = 0.473, *p* = 0.004), confirming the uric acid-SNQ correlation.

### 3.2 Subgroup analysis

The results of the preliminary analysis revealed a significant correlation between uric acid and SNQ values. To consolidate our findings, we designed a subgroup analysis using the available data. This study enrolled 60 patients aged 20–50 years (mean 36 years), all with high physical activity demands in daily life. Subsequent subgroup analysis categorized patients into two groups based on diagnostic criteria for hyperuricemia: Hyperuricemia group (33 patients): Serum UA > 420 μmol/L (male) or >360 μmol/L (female); Normal UA group (27 patients): UA ≤ diagnostic thresholds. This standard is formulated in accordance with the National Health Commission of the People’s Republic of China (2024 edition Guidelines). The two groups were matched for age, BMI, and gender to ensure baseline comparability. All baseline demographic characteristics are summarized in Table 3.

**Table 3:** Demographics of patients.

Characteristic	Normal group (*n* = 27)	Hyperuricemia group (*n* = 33)	*p*-value
Age (years)	36.4 ± 9.6	36.4 ± 6.1	0.982
Height (cm)	174.3 ± 4.4	175.0 ± 6.7	0.627
Weight (kg)	77.0(82.0–73.0)	80.0(84.0–74.0)	0.311
BMI (kg/m^2^)	25.5(26.7–23.9)	25.6(26.9–24.7)	0.547
Sex			0.198
Male	25 (92.6%)	33 (100.0%)	
Female	2 (7.4%)	0 (0.0%)	
Affected side			0.916
Right	11 (40.7%)	13 (39.4%)	
Left	16 (59.3%)	20 (60.6%)	

**Figure 5:**
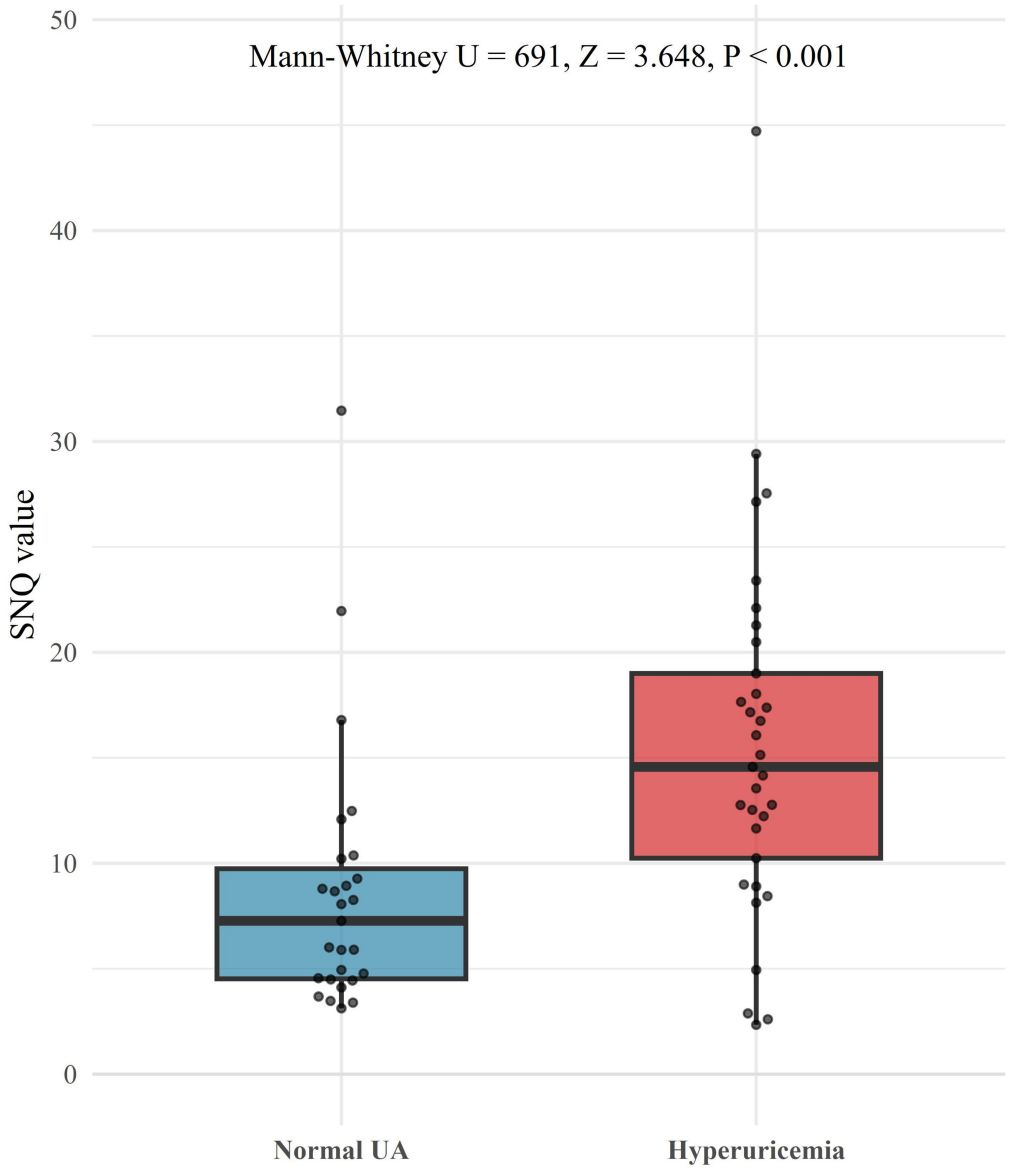
Box plot comparing SNQ values between the normal UA group and the hyperuricemia group. The difference was statistically significant (Mann–Whitney U test, *P* < 0.001).

The Mann–Whitney U test revealed a statistically significant difference in SNQ values between the two groups (*U* = 691.0, *Z* = 3.648, *p* < 0.001). Patients with hyperuricemia exhibited markedly higher median SNQ values (14.57) compared to the normouricemia group (7.27), indicating poorer early-stage healing in individuals with high UA levels compared to the normal people. The boxplot is shown in Figure 5. This finding aligns with the preliminary analysis results, reinforcing the robustness of UA as a predictor of delayed healing.

## 4 Discussion

This study provides novel evidence indicating that hyperuricemia is associated with delayed postoperative healing of Achilles tendon rupture. Both Spearman’s rank correlation analysis and multivariate linear regression results strongly support serum uric acid (UA) as an independent predictor of delayed tendon repair. To consolidate our findings, subsequent subgroup analysis demonstrated statistically significant differences in SNQ values between the hyperuricemia group and the normal group. Furthermore, with the numbers available, no significant difference could be detected between lipid metabolism markers (e.g., total cholesterol, triglycerides) and SNQ in this study. However, the results of this study showed that the Spearman correlation coefficient between serum uric acid and SNQ was *ρ* = 0.400, indicating a weak-to-moderate positive correlation. Achilles tendon healing is governed by a combination of factors such as the local mechanical environment, systemic metabolic status, and rehabilitation adherence. Therefore, a single metabolic marker cannot explain most of the variability in healing outcomes. Nevertheless, this association is genuine rather than spurious. Therefore, uric acid has limited predictive value and should only be used as an adjunctive risk predictor. Patients with hyperuricemia should be considered a potential high-risk group for poor healing, but treatment decisions should not be made solely based on uric acid levels.

Hyperuricemia is a metabolic disorder characterized by abnormal purine metabolism. Substantial evidence confirms hyperuricemia as a risk factor for Achilles tendon rupture (ATR). A case–control study by Jiang et al. demonstrated that hyperuricemic patients have a 2.036-fold increased risk of ATR compared to normouricemic individuals (OR = 2.036, 95% CI = 1.400–2.961, *p* < 0.001) (6). Similarly, Chen et al. identified smoking, elevated BMI, total cholesterol (TC), and uric acid (UA) as risk factors for ATR, proposing that adjunctive tests for TC and UA in blood biochemistry may be helpful in predicting the risk of ATR (5). Animal experiments reveal that UA may impair tendon function by disrupting the normal activity of tendon stem/progenitor cells (TSPCs) (7). TSPCs play a critical role in the tendon healing process. During the inflammatory phase of tendon healing, TSPCs participate in regulating inflammatory responses and suppressing excessive scar formation and fibrosis, thereby promoting tendon repair (8, 9). Additionally, TSPCs and their exosomes accelerate tendon cell proliferation, enhance collagen synthesis, and balance extracellular matrix (ECM) remodeling (10). The tendon ECM is primarily composed of abundant type I collagen with smaller amounts of types III and IV collagen and proteoglycans (6), The remodeling of the ECM significantly influences the mechanical strength and biomechanical function of tendons. In a rat tendon injury model, Wang et al. found that TSPCs and their exosomes markedly enhance collagen fiber expression and increased biomechanical properties of the ultimate stress and maximum loading of healed tendons (10, 11).

Importantly, hyperuricemia may impair these repair mechanisms. Experimental studies by Liang et al. revealed that uric acid induces dose-dependent cytotoxic effects on rat TSPCs, suppressing tendon collagen synthesis while markedly upregulating the transcription of matrix degradative enzymes and proinflammatory factors (7). This dual pathway—combining accelerated ECM breakdown and sustained inflammatory signaling—likely underlies the compromised tendon healing observed in hyperuricemia. Furthermore, hyperuricemia-driven deposition of monosodium urate (MSU) crystals within tendon tissues activates the NLRP3 inflammasome (4), triggering excessive release of IL-1β and IL-18, which perpetuates a pro-inflammatory microenvironment detrimental to tissue regeneration (12, 13). Collectively, these findings position hyperuricemia as an important contributor to delayed Achilles tendon repair. Nevertheless, deeper mechanistic insights into UA-associated cellular pathways await further cell-level investigations.

The Signal-to-Noise Quotient (SNQ) is a validated quantitative MRI biomarker for assessing tendon maturation and repair quality, extensively used in evaluating postoperative healing of ligaments including the anterior cruciate ligament (ACL) and anterior talofibular ligament (ATFL) (14, 15). This study applies SNQ methodology to Achilles tendon repair, leveraging its established capacity to objectively quantify microstructural tissue properties through T2-weighted signal intensity analysis. Unlike conventional clinical scores (e.g., AOFAS, ATRS) that primarily measure functional recovery, SNQ detects early microstructural changes by analyzing water content and collagen organization, enabling identification of subclinical healing abnormalities (16) The methodology’s clinical validity is reinforced by its proven correlation with rerupture risks in tendon repair studies (17), and its predictive value in preoperative MRI assessments of repair quality (18). In this study, we used the signal-to-noise quotient (SNQ) derived from conventional T2-weighted imaging as a marker of Achilles tendon healing. However, we acknowledge that quantitative MRI sequences, such as T2 mapping and T2* mapping, have recently emerged as more direct and sensitive tools for assessing tissue microstructure (19). T2 mapping can reflect changes in tissue water content and the organizational integrity of collagen fibers, and its T2 values have been shown to correlate significantly with histological changes during Achilles tendon healing. T2 mapping has been demonstrated, in a rabbit Achilles tendon transection model, to be capable of noninvasively reflecting the biological process of tendon healing (20, 21). Although the retrospective design of this study did not allow the use of these sequences, future prospective studies may consider incorporating T2 mapping or T2* mapping for a more precise assessment of Achilles tendon healing quality.

This study demonstrates significant clinical relevance by highlighting a potential association between uric acid metabolism and delayed postoperative recovery following Achilles tendon repair, suggesting that preoperative serum uric acid evaluation could be integrated into clinical assessments to guide personalized rehabilitation protocols. Future randomized controlled trials (RCTs) can investigate urate-lowering interventions in hyperuricemic ATR cohorts to determine whether perioperative UA control enhances tendon repair outcomes. However, this study has several limitations. First, the study is a single-center retrospective investigation with a relatively small sample size. Second, our analysis predominantly examined the influence of metabolic factors on early postoperative healing (3-month follow-up) of the Achilles tendon, while extended long-term follow-up data and functional outcome data remain absent in the current dataset. The implications of findings on long-term functional outcomes require further validation through large-scale cohort studies. Third, the sex distribution of the enrolled patients was imbalanced, with only two females among the 60 patients. Therefore, the generalizability of our conclusions to female patients should be cautious. Future studies should enroll patients with a more balanced sex ratio to verify whether sex differences exist.

## 5 Conclusion

Hyperuricemia is a significant metabolic factor impairing early ATR healing, highlighting the clinical utility of preoperative UA screening for personalized rehabilitation. Perioperative uric acid control is recommended for hyperuricemic patients to optimize Achilles tendon healing outcomes.

## Data Availability

The original contributions presented in the study are included in the article/Supplementary material, further inquiries can be directed to the corresponding author.
