# Supplementary material for: Correlation analysis of metabolic parameters influencing postoperative tendon healing after Achilles tendon rupture repair
Source: Front Med (Lausanne). 2026 Jun 30;13:1829337. doi: 10.3389/fmed.2026.1829337 (PMC13364573; doi:10.3389/fmed.2026.1829337)
Supplement: Supplementary file 1 [file Table_1.docx]

Supplementary Table S1. Comparison of baseline characteristics between complete-case and missing groups

| Variable | | Complete (n=45) | Missing (n=15) | P value |
| --- | --- | --- | --- | --- |
| Age (years) | | 38 [34-41] | 34 [30-36.5] | 0.016 |
| BMI (kg/m²) | 25.6 [24.6-26.6] | | 24.3 [23.7-27.2] | 0.294 |
| Uric acid (μmol/L) | 429 [341-468] | | 464 [394-506] | 0.064 |
| SNQ | 10.2 [5.9-16.8] | | 12.8 [6.6-19.7] | 0.432 |
| Total bilirubin (μmol/L) | 16.9 [13.4-19.4] | | 16.4 [12.4-20.1] | 0.824 |
| Male sex | 43 (95.6%) | | 15 (100%) | 1.000 |
